# Bilateral pulmonary vein stenting for treatment of massive hemoptysis caused by pulmonary vein stenosis following catheter ablation for atrial fibrillation

**DOI:** 10.1186/s12872-019-1141-0

**Published:** 2019-07-04

**Authors:** Dong Yu, Bing Jie, Ling-Ling Li, Sen Jiang

**Affiliations:** Department of Radiology, Shanghai Pulmonary Hospital, Tongji University School of Medicine, 507 Zhengmin Road, Shanghai, 200433 China

**Keywords:** Atrial fibrillation, Pulmonary vein stenosis, Radiofrequency ablation, Hemoptysis, Endovascular stent

## Abstract

**Background:**

Massive hemoptysis is a life-threatening condition. Massive hemoptysis caused by pulmonary vein stenosis (PVS) after radiofrequency catheter ablation for atrial fibrillation (AF) is rare. However, bilateral lung hemorrhage following bilateral PVS is extremely rare.

**Case presentation:**

We herein describe a 62-year-old man with refractory massive hemoptysis after radiofrequency catheter ablation for AF, which was successfully controlled by surgical lobectomy and endovascular bilateral PV stenting. The hemorrhage was derived from the bilateral lungs following PV obstruction and bilateral PVS, which was definitively diagnosed by bronchoscopic examination. The patient had no recurrence of hemoptysis during a follow-up period of 30 months, and the PV stents had not narrowed as shown by computed tomography 30 months after stent placement.

**Conclusions:**

Massive hemoptysis can be caused by bilateral PVS after radiofrequency catheter ablation for AF, and hemorrhage from the bilateral lungs in such patients is extremely rare. Nevertheless, cardiologists, interventional radiologists, and pulmonologists should consider the potential for massive hemoptysis caused by PVS.

**Electronic supplementary material:**

The online version of this article (10.1186/s12872-019-1141-0) contains supplementary material, which is available to authorized users.

## Background

Massive hemoptysis is a major medical emergency with a wide range of underlying causes. The major sources are the bronchial and non-bronchial systemic arteries, and the least common source is the pulmonary artery [1, 2]. Pulmonary vein stenosis (PVS) can also lead to hemoptysis, although the clinical findings are nonspecific and often misleading [3–8]. Endovascular systemic artery embolization is deleterious for management of PVS-induced hemoptysis [3]. Life-threatening hemoptysis caused by PVS is rare, while that caused by bilateral PVS is extremely rare. We herein present a case of refractory massive hemoptysis caused by PVS after radiofrequency catheter ablation for atrial fibrillation (AF), which was treated by endovascular bilateral PV stent placement.

## Case presentation

**Fig. 1:**
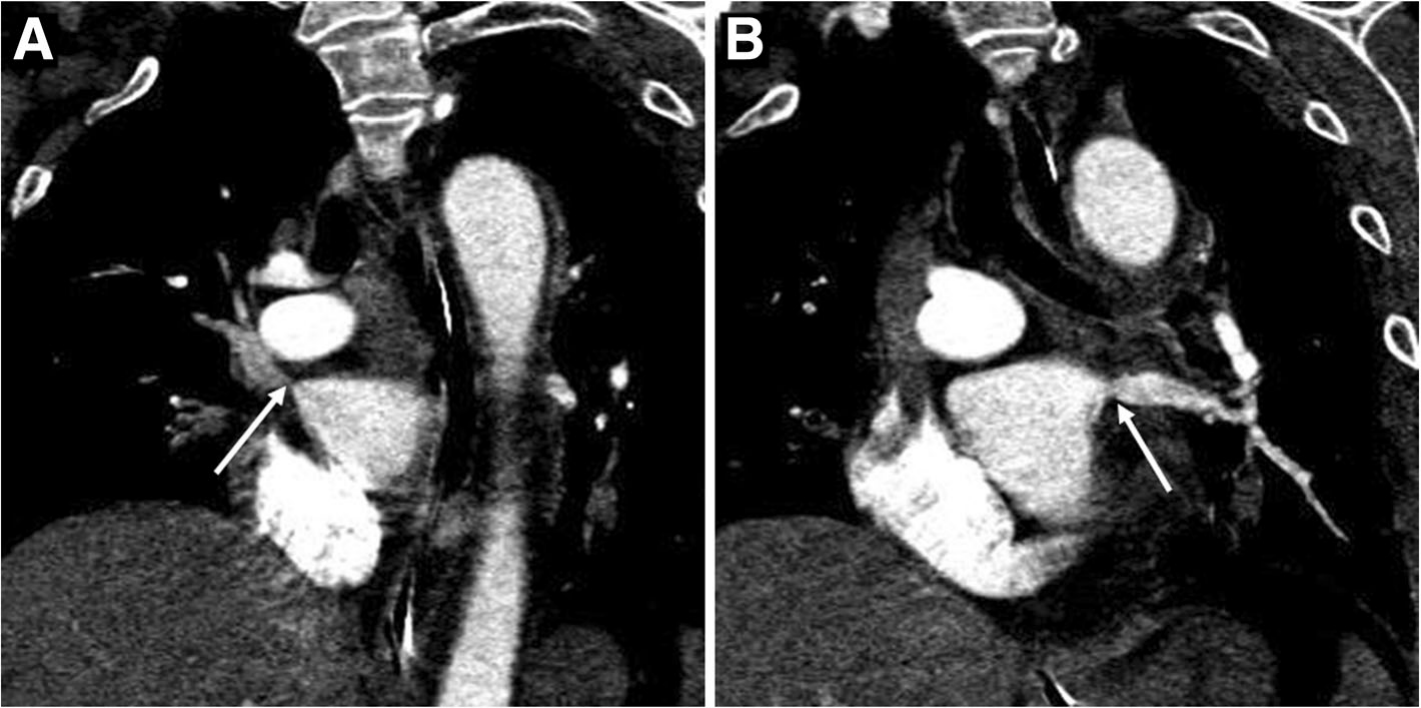
**a** Oblique coronal maximum intensity projection computed tomography (CT) showing stenosis (approximately 95%, *arrow*) of the right superior pulmonary vein (PV). **b** Oblique coronal maximum intensity projection CT showing stenosis (approximately 90%, *arrow*) of the left inferior PV

A 62-year-old man was admitted to our hospital in March 2016 for control of massive hemoptysis of unknown cause. He had experienced hemorrhage for 10 consecutive days (maximum of 800 mL/day) despite conservative intravenous therapy and two bronchial artery embolization procedures at a local hospital. Upon admission to our hospital, the chest computed tomography (CT) findings obtained at his local hospital revealed complete obstruction of the left superior PV and stenosis of the right superior and left inferior PV, and bronchoscopic examination revealed hemorrhage from the left upper lobe. His medical history included radiofrequency catheter ablation for AF 5 months previously. Surgical left upper lobectomy was performed on an emergency basis and the hemoptysis was controlled for 2 days. However, on postoperative day 3, he developed another episode of massive hemoptysis (hemorrhage of 500 mL). Physical examination revealed severe moist rales over the bilateral thorax. His hemoglobin level was 73 g/L. The patient underwent bronchoscopic examination and multidetector row CT angiography. The bronchoscopic examination revealed hemorrhage from both the right upper lobe and left lower lobe. CT angiography revealed stenosis in the right superior PV (approximately 95%) and left inferior PV (approximately 90%) (Fig. 1). When offered urgent surgical venoplasty or nonsurgical PV stenting, the patient chose minimally invasive catheter-guided PV stent implantation. Bilateral PV stenting was therefore performed to control refractory massive hemoptysis.

**Fig. 2:**
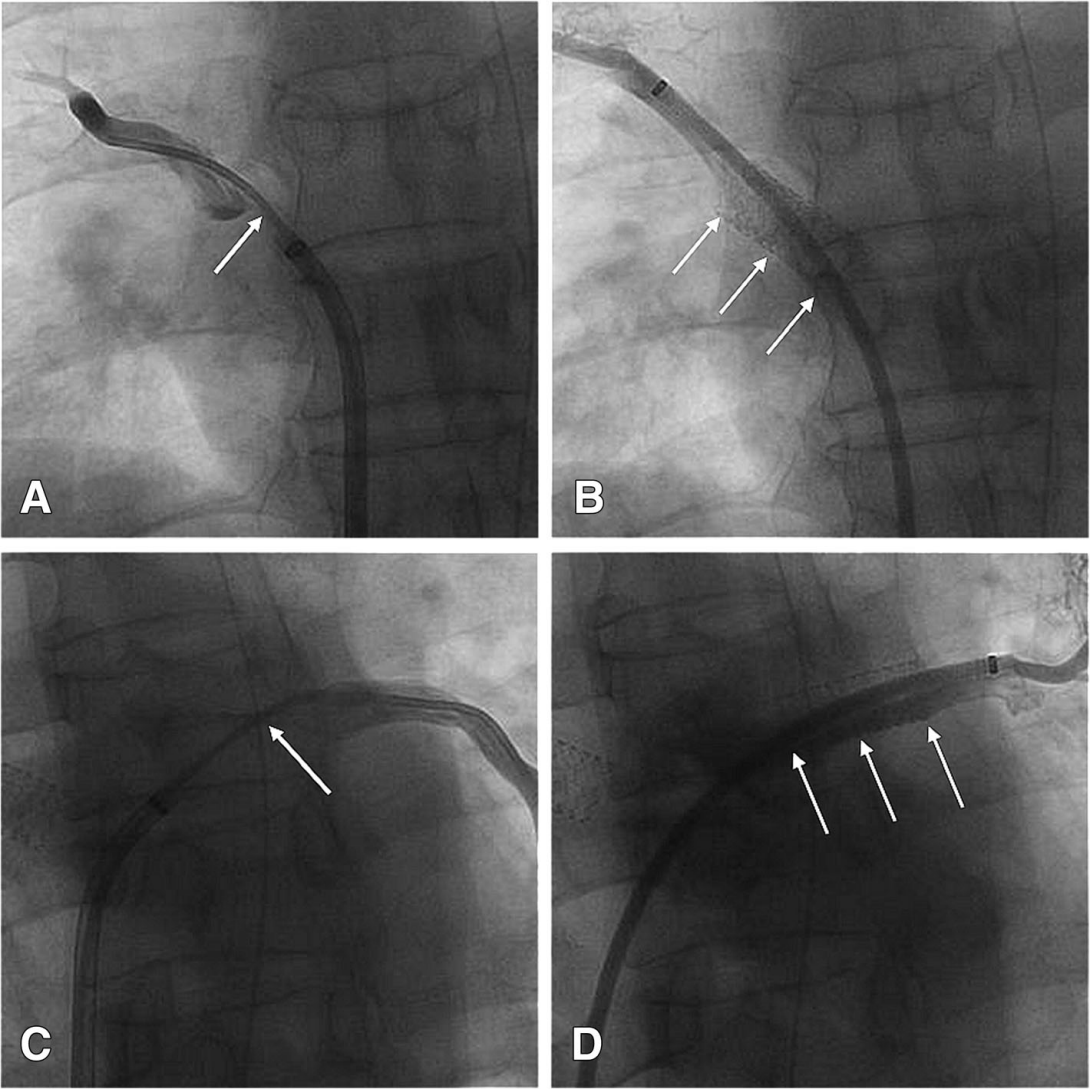
**a**, **c** Selective angiography of the right superior and left inferior PV with an H1 catheter (*arrow*) showing severe narrowing of the two PVs. **b**, **d** Selective angiography following deployment of the stents (*arrows*), with restoration of normal sheath diameter

Procedural access was obtained through the right femoral vein, and a 12-Fr venous sheath (Cook Medical, Bloomington, IN, USA) was placed. Selective right upper, right lower, and left lower lobe pulmonary angiography was performed using a 4-Fr H1 catheter (Cordis Corp., Miami Lakes, FL, USA) to indirectly evaluate the location and narrowing of the PVs. An 8.5-Fr sheath (SL 1™; St. Jude Medical, Saint Paul, MN, USA) with a Brockenborough™ needle (St. Jude Medical) was used to cross the intra-atrial septum, through which direct right superior and left inferior pulmonary venography was performed using a 4-Fr H1 catheter (Cordis Corp.). Venography showed severe PV narrowing (Fig. 2a, c; Additional file 1: Video S1, Additional file 2: Video S3). From the catheter, a 260-cm Amplatzer guidewire (Cordis Corp.) was exchanged, and a 90-cm 7-Fr sheath (Flexor Check-Flo Introducer sheath; Cook Medical) was inserted into the distal side of the PVS. From the sheath, a 10- × 25-mm and a 9- × 25-mm balloon-expandable stent (Express™ LD Vascular; Boston Scientific, Marlborough, MA, USA) were placed in the right superior and left inferior PVs, respectively (Fig. 2b, d; Additional file 3: Video S2, Additional file 4: Video S4). The PV pressure was measured before and after PV stent placement. The pressure of the right superior PV (systolic/diastolic/mean) decreased from 44/23/32 to 16/3/10 mmHg and the pressure of the left inferior PV (systolic/diastolic/mean) decreased from 28/17/22 to 15/6/10 mmHg, respectively. The proximal part of two stents extended to left atrium about 8 mm.

**Fig. 3:**
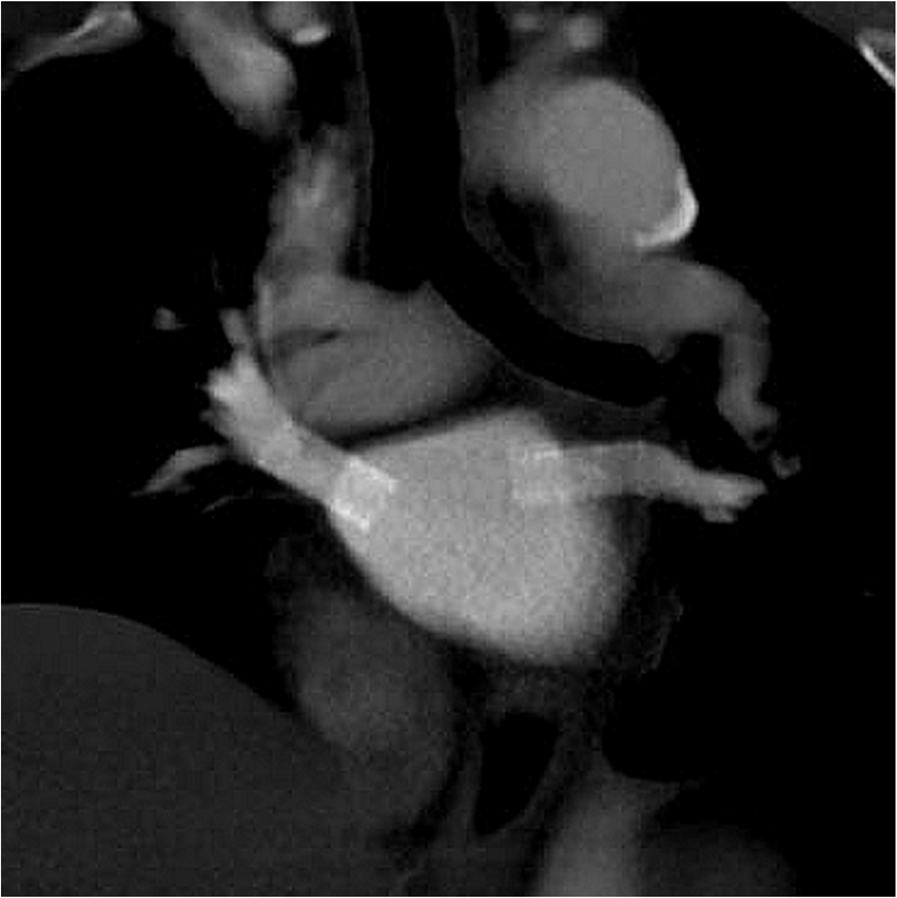
Oblique coronal maximum intensity projection CT showing no restenosis of the two PVs at 30-month follow-up

This procedure resulted in control of the hemoptysis, and the patient had developed no further episodes of hemoptysis by the 30-month follow-up. During stent placement and after the procedure, anticoagulation or anticoagulant was continued. Before the procedure, 5000 IU of intravenous heparin was administered. Immediately after the procedure, the patient received an anticoagulant agent (low-molecular-weight heparin sodium), and this treatment was continued until discharge. After discharge, the patient received an oral anticoagulant agent (warfarin) and antiplatelet agent (clopidogrel bisulfate) until the 12-month follow-up CT examination, which showed widening of the PV stents. The 30-month follow-up CT examination revealed no restenosis (Fig. 3).

## Discussion and conclusions

Both the pulmonary and bronchial circulations is drained via the PVs into the left atrium. PVS causes blockade of the pulmonary and bronchial circulation return with development of PV hypertension. Depending on the acuity of the PVS or PV obstruction and the development of venous collaterals, venous parenchymal and mucosal bleeding is usually darker than hemoptysis of the systemic arteries [3]. The causes of PVS may be congenital, acquired, or functional; among these, acquired PVS is the most common [3]. The causes of acquired PVS include mediastinal or pulmonary masses resulting in extrinsic compression, granulomatous fibrosing mediastinitis, complications of catheter ablation for AF and lung/heart surgery, and intraluminal growth of neoplasms in the PV or left atrium [3].

AF is one of the most common cardiac rhythm disorders. The pathophysiological origin is predominantly in the PV, making it treatable by ablative catheter procedures [4]. PVS is a known complication of ablation inside or near the PV. The incidence of PVS has decreased substantially with improvements in techniques, and is now estimated to complicate between 0.3 and 3.4% of AF ablation procedures [8]. Some patients with PVS are asymptomatic. Furthermore, PVS symptoms can include cough, dyspnea, chest pain, or hemoptysis, all of which are consistent with bronchitis, asthma, pneumonia, and pulmonary embolism [4–8]. However, acute massive hemoptysis caused by PVS after radiofrequency catheter ablation for AF is rare. In the present case of acute massive hemoptysis, the hemorrhage was derived from the bilateral lung following bilateral PVS, which was definitively diagnosed by bronchoscopic examination.

Surgical lobectomy or pneumonectomy (such as the initial treatment in our case) can be a life-saving procedures performed for complete PV occlusion and acute massive hemorrhage [3]. Surgical therapeutic options for management of patients with hemoptysis caused by acquired PVS include sutureless venoplasty or pericardial patchplasty [3]. Nonsurgical therapeutic options for management of patients with hemoptysis caused by acquired PVS include invasive balloon angioplasty and stent implantation [3–8]. Stent implantation is superior to balloon angioplasty, although high rates of restenosis have been reported for both procedures (47–72%) [4–8]. Stenting for PVS is usually performed with a balloon-expandable stent of approximately 8- to 12-mm diameter [4–8]. In our case, 9 and 10-mm balloon-expandable stents were placed in the right superior and left inferior PV, respectively, with no restenosis at the 30-month follow-up CT examination. The proximal part of the stents reached far into the left atrium cavity in our case and anticoagulation should be continued after the procedure to prevent thrombosis. Yamauchi et al. [9] reported a case of selective pulmonary artery occlusion to treat hemoptysis associated with PV obstruction; this treatment also has potential for treating hemoptysis caused by a PV tumor thrombus.

In conclusion, we have herein described an extremely rare case of massive hemoptysis caused by bilateral PVS after radiofrequency catheter ablation for AF and hemorrhage from the bilateral lungs. Nevertheless, cardiologists, interventional radiologists, and pulmonologists should consider the potential for massive hemoptysis caused by PVS.

## Additional files

Additional file 1: : **Video S1.** Selective angiography of the right superior PV. (AVI 4278 kb)

Additional file 2: : **Video S3.** Selective angiography of the right superior PV following deployment of the stent. (AVI 4460 kb)

Additional file 3: : **Video S2.** Selective angiography of the left inferior PV.(AVI 3546 kb)

Additional file 4: : **Video S4.** Selective angiography of the left inferior PV following deployment of the stent. (AVI 3045 kb)

## Data Availability

Not applicable.

## Abbreviations

AF: Atrial fibrillation
CT: Computed tomography
PV: Pulmonary vein
PVS: Pulmonary vein stenosis
